# Pharmacological Properties and Molecular Targets of Alisol Triterpenoids from *Alismatis* Rhizoma

**DOI:** 10.3390/biomedicines10081945

**Published:** 2022-08-11

**Authors:** Christian Bailly

**Affiliations:** OncoWitan, Scientific Consulting Office, 59290 Lille (Wasquehal), France; christian.bailly@oncowitan.com

**Keywords:** *Alismatis* rhizoma, alisol, cancer, inflammation, molecular targets, pharmacology, protostane triterpenoids

## Abstract

More than 100 protostane triterpenoids have been isolated from the dried rhizomes of *Alisma* species, designated *Alismatis* rhizoma (AR), commonly used in Asian traditional medicine to treat inflammatory and vascular diseases. The main products are the alisols, with the lead compounds alisol-A/-B and their acetate derivatives being the most abundant products in the plant and the best-known bioactive products. The pharmacological effects of Ali-A, Ali-A 24-acetate, Ali-B, Ali-B 23-acetate, and derivatives have been analyzed to provide an overview of the medicinal properties, signaling pathways, and molecular targets at the origin of those activities. Diverse protein targets have been proposed for these natural products, including the farnesoid X receptor, soluble epoxide hydrolase, and other enzymes (AMPK, HCE-2) and functional proteins (YAP, LXR) at the origin of the anti-atherosclerosis, anti-inflammatory, antioxidant, anti-fibrotic, and anti-proliferative activities. Activities were classified in two groups. The lipid-lowering and anti-atherosclerosis effects benefit from robust in vitro and in vivo data (group 1). The anticancer effects of alisols have been largely reported, but, essentially, studies using tumor cell lines and solid in vivo data are lacking (group 2). The survey shed light on the pharmacological properties of alisol triterpenoids frequently found in traditional phytomedicines.

## 1. Introduction

**Figure 1:**
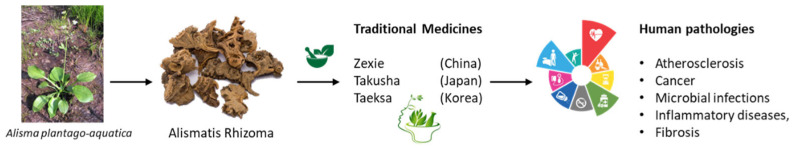
Medicinal uses of *Alismatis* rhizoma. The dried rhizomes of the plant *Alisma plantago-aquatica* are used to prepare the traditional medicinal products in Asian countries and the medicine is used to treat different pathologies, as indicated.

The dried rhizomes of the plants *Alisma orientale* Juzepczu and *Alisma plantago*-*aquatica* (Alismataceae), commonly referred to as *Alismatis* rhizoma, have long been used in folk medicine to treat a variety of diseases, including hyperlipidemia, diabetes, bacterial infection, edema, oliguria, diarrhea, dizziness, and certain inflammatory diseases. The phytomedicine is known as Takusha in Japanese, and Taeksa in Korean and Zexie in Chinese (Figure 1). In China, *Alismatis* rhizoma (Zexie) can be further separated into “Chuan Zexie” (Sichuan and Hubei provinces), “Jian Zexie” (Fujian and Jiangxi provinces), and “Guang Zexie” (Guangxi province) according to the producing areas [1].

**Figure 2:**
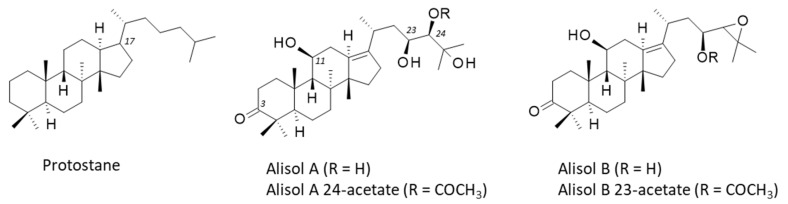
Structures of the protostane terpenoid skeleton and the main alisol compounds, Ali-A/-B and their acetate derivatives. The numbering of specific positions (discussed in the text) is indicated.

*Alismatis* rhizoma is known to contain a large diversity of bioactive components, mainly terpenoids endowed with anti-inflammatory, antioxidant, and antimicrobial activities. A recent phytochemical survey has identified over 220 bioactive molecules, including di-, tri-, and sesqui-terpenoids, alkaloids, phenylpropanoids, flavonoids, steroids, and polysaccharides [2]. The main subgroup of triterpenes found in *Alismatis* rhizoma are the alisols, a family of 22 compounds designated alisol A to alisol V [3,4]. These compounds are tetracyclic protostane-type triterpenoids, with a variable side chain at position 17 (Figure 2). Hydroxyl groups can be present at positions 11, 23, and 24, eventually acetylated or implicated in an epoxide function. The protostane triterpene skeleton is relatively rare in nature [5]. In *A. orientale*, its biosynthesis is regulated by methyl jasmonate [6]. The leading compounds in the series are alisol A and its epoxide derivative alisol B (Figure 2) which have been largely studied for their diverse pharmacological effects. Both Ali-A and Ali-B display marked anti-inflammatory and anti-proliferative properties, useful for the treatment of diverse human pathologies, including metabolic disorders and cancers. In the present review, I have analyzed the main pharmacological properties of these bioactive natural products, and their known or proposed molecular targets. The pharmacology of these interesting natural products is discussed.

## 2. TCM Made from *Alismatis* Rhizoma

*Alismatis* rhizoma (AR) is commonly used in traditional Chinese medicine (TCM). The two *Alisma* species which correspond to AR are largely cultivated, mainly in Fujian, Jiangxi, and Sichuan provinces (China). The rhizosphere microbiomes for the two plants are slightly distinct, with specific bacteria (such as Steroidobacter, Pseudolabrys, Nevskia, and Nitrospira) which are considered important for the biosynthesis and accumulation of protostane triterpenes in the plant tubers [7]. *Alismatis* rhizoma is listed in the 2020 edition of the Chinese Pharmacopoeia, with specific recommendations on quality standards [8]. Those rhizoma are included in several TCMs, such as Ye Tianshi used for the treatment of dampness and heat [9], Zexie Tang used to treat metabolic diseases and liver injuries [10], Liuwei Dihuang decoction used to improve kidney yin deficiency [11], Hugan Qingzhi formula which is used to treat chronic liver diseases [12], Gandou decoction used to treat Wilson’s disease [13,14], and Dangguishaoyao-san (also called Toki-shakuyaku-san or TJ-23) used to reduce the symptoms of Alzheimer’s disease [15,16]. *Alismatis* rhizoma is also present in different TCM prescriptions, such as Qi-Li-Qiang-Xin capsule used for the treatment of chronic heart failure [17,18] and Qing-Fei-Pai-Du which is a lung cleansing and detoxifying decoction used historically for the treatment of exogenous fever, and recently proposed to reduce symptoms associated with coronavirus disease 2019 (COVID-19) [19]. The product is also used in other traditional medicines, notably in traditional Japanese medicine (Kampo) with the preparation called Hachimijiogan which is used to alleviate anxiety disorders in patients with dementia [20], and in Korean medicine with the herbal medicine prescription called Oreongsan (also known as Goreisan in Japanese and Wulingsan in Chinese), containing AR, and used to treat symptoms such as edema, hangover, and diarrhea [21,22]. Another Korean prescription called Geumgwesingihwan is a traditional herbal product composed of 8 medicinal herbs, including *Alisma plantago-aquatica,* and contains Ali-B acetate [23]. I can also mention the Japanese herbal medicine Sairei-to (TJ-114, or Chai-Ling-Tang in Chinese) used to reduce lower urinary tract symptoms. Sairei-to is made from 12 medicinal herbs including AR [24,25]. There are also composite products based in part on AR, such as Yirui capsules, sold in health food stores, to improve the blood lipid state in patients with hyperlipidemia and to reduce the risk of atherosclerosis [26,27]. It is certain, AR is an essential component of Asian traditional medicine.

## 3. *Alismatis* Rhizoma Extracts: Phytoconstituents and Pharmacological Effects

Analytical methods have been developed to define the composition of *Alismatis* rhizoma (AR) extracts and to provide a quality control assessment for the preparations [28]. The triterpene composition can vary according to the geographic origin of the plant tubers, but the main compounds are always Ali-A and Ali-B [29]. A monitoring of the botanical source of AR is important, as it defines the phytochemical content of the extracts [2,30]. Crude AR extracts contain a multitude of triterpenoids and their composition also varies with the processing method. In a recent study, up to 114 triterpenes were identified from salt-processed and bran-processed AR extracts using state-of-the-art ultra-high performance liquid chromatography coupled with mass spectrometry [31]. Another study pointed out over 220 compounds, including di-/tri-/sesqui-terpenoids, alkaloids, phenylpropanoids, flavones, steroids, and polysaccharides from AR extracts [2]. New compounds are regularly identified from AR extracts, notably novel anti-inflammatory agents [32]. Once administered, these triterpenes are metabolized, thus producing additional new compounds and derivatives. More than 230 transformed constituents have been mentioned recently from orally administered AR extracts [33]. However, in all cases, the main bioactive compounds are the alisols, in particular Ali-A/-B/-C and their acetate derivatives [34]. Of course, other products can be found in AR extracts depending on the process used. For example, an essential oil from tubers of *A. orientale* can be specifically prepared, rich in aroma-active volatile compounds [35].

Extracts of *Alismatis* rhizoma are commonly used in China to lower cholesterol and as an anti-inflammatory agent. The TCM product is recommended for excreting dampness caused by a spleen deficiency, which relies on a diuretic effect. This effect has been well characterized when using an ethanol extract of AR, but not with an aqueous extract. In fact, the ethanolic extract displays a dual effect on renal function, promoting the diuretic activity at lower doses and inhibiting diuretic activity at higher doses [36]. The diuretic effect is largely supported by the alisol compounds [37]. RA extracts display a range of pharmacological effects, not limited to the induction of diuresis. They also present hepatoprotective, immunomodulatory, anti-osteoporotic, anti-inflammatory, antitumor, antibacterial, and antiviral activities, as described in previous comprehensive reviews [2,38,39]. AR extracts are considered to be safe and well tolerated in humans. A toxicology assessment of AR aqueous extracts in rats revealed no specific target organs and the oral no-observed-adverse-effect level (NOAEL) was >2 g/kg/day [40]. However, an evaluation with an ethanol extract pointed out a risk of chronic toxicity in kidneys when using a high dosage of the AR ethanol extract for an extended period [41]. The nephrotoxicity effect has been ascribed to the terpenoids found in those extracts and capable of inducing cell death (apoptosis) of kidney proximal tubular cells [42]. This is a paradoxical effect because the Chinese medicine Wuling San, which is made from AR and four other plants, is used to promote kidney function and diuresis. Wuling San has demonstrated a renal protective effect by modulating renal organic ion transporters in hyperuricemic mice [43]. In addition, a combination of the two Chinese herb decoctions, AR and *Smilacis glabrae* rhizoma, has been shown to reduce hyperuricemia and kidney damage in a rat model [44]. The nephrotoxicity reported in some studies with AR extracts may be attributed to specific compounds, such as alisol C, 16, 23-oxido-alisol B, and alisol O, which are considered more toxic than Ali-A and Ali-B [45].

## 4. Alisols and Related Terpenoids

**Figure 3:**
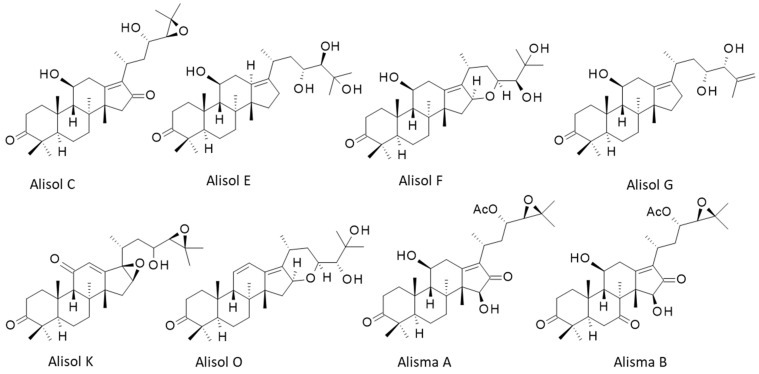
Structures of 8 selected alisol derivatives. The series includes 22 compounds (Ali-A to Ali-V) and 100 derivatives.

In the plant, protostanes are biosynthesized from squalene with the support of squalene epoxidases regulated by methyl jasmonate [6,46]. There are 22 alisol compounds, designated alisol A (Ali-A) to alisol V (Ali-V), and a multitude of related compounds bearing an acetate group at position 23 or 24 of the side chain appended to the tetracyclic protostane unit. There are also derivatives with a modified side chain at C-17, equipped with an open or epoxy aliphatic side chain, with a fused ring at C-16/C-17 or with a modified protostane unit (nor- or seco-protostane). The series includes numerous derivatives, such as the alismanols, alismanins, alisolides, and others. In 2020, Wang and co-workers had identified more than 115 protostane-type terpenoids from *Alisma* species [47]. Here, we will essentially refer to Ali-A, -B, and -C, and occasionally to a few other compounds mentioned in Figure 2 and Figure 3. Most of them preserve the C-3 carbonyl group of characteristics of Ali-A and a highly oxygenated C-17 side chain. New protostane terpenoids are regularly identified and isolated from AR, taking advantage of novel, highly efficient, and precise analytical methods. For example, the two new compounds, called Alisma A and Alisma B, have been recently discovered using a new analytical methodology called ‘force iteration molecular designing’. With this approach, the authors identified 473 protostane terpenoids from the two plant species of AR [4]. Here, we will mainly focus on Ali-A and Ali-B to evoke their pharmacological effects and molecular targets. The different properties will be evocated in turn. The first three alisols, -A, -B, and -C, and their monoacetate derivatives were isolated in 1970 by researchers from Takeda Chemical Industries Ltd. (Osaka, Japan), and they also rapidly characterized the hypocholesterolemic activity of the products [48,49]. More than 50 years later, alisol A remains an interesting natural product mainly investigated for its multiple properties, and the knowledge gained from it has been utilized for hundreds of analogous natural products.

## 5. Pharmacological Properties of Alisols

### 5.1. Lipid-Lowering Effects and Hepatoprotection

Historically, in Asia, AR has been known for its diuretic action. It was traditionally used to treat diabetes and swelling. A search of AR extracts for compounds with a hypocholesterolemic action was initiated in the late 1960s, leading to the identification of active fractions and then pure compounds. Ali-A/-B/-C, along with their monoacetates, were discovered to be capable of reducing plasma and liver cholesterol levels in rats after oral administration [49]. The mode of action for these compounds has been investigated much more deeply now, leading to a better understanding of the pathways implicated in the capacity of Ali-A/-B to reduce lipid accumulation and lipotoxicity. A recent work demonstrated how Ali-B regulates hepatic gene expression via the RARα-HNF4α-PPARγ cascade in a murine model of non-alcoholic steatohepatitis (NASH). Specifically, Ali-B enhances *RARα* gene expression, thereby reducing expression of HNF4α and PPARγ, leading to a suppressed expression of translocase fatty acid CD36 [50]. The lipid-lowering components of AR includes Ali-B, Ali-B 23-acetate, and Ali-C 23-acetate, which are believed to bind to the farnesoid X receptor (FXR) protein and to behave as agonists, according to a molecular docking study [51]. The hypothesis is entirely plausible because Ali-B 23-acetate has been shown to increase the expression of FXR in cultured hepatocyte L02 cells and to activate hepatic BSEP (bile salt export pump) signaling [52]. The capacity of Ali-B 23-acetate to function as a FXR agonist has been demonstrated in a model of renal ischemia-reperfusion injury. The compound was found to activate renal FXR and to induce *FXR* downstream gene expression in mouse kidneys [53], and also in a model of non-alcoholic steatohepatitis (NASH) [54]. It is a potent agonist of several pregnane X receptors (PXRs), including FXR, but also LXR and PPARα/δ/γ [55]. This compound displays marked hepato-protective functions, due to FXR-mediated gene regulation [56,57,58,59]. The related compound, Ali-A 24-acetate, is also able to reduce liver lipid deposition in hyperlipidemic mice via a blockade of AMPK activation to promote glucose metabolism and by promoting expression of ATP binding cassette transporters ABCG1 and ABCA1 [60,61]. The compound stimulates lipolysis of adipocytes by activating protein kinase A (PKA)-mediated phosphorylation of hormone-sensitive lipase [62]. These observations make Ali-A 24-acetate and Ali-B 23-acetate good candidates for the treatment of nonalcoholic fatty liver disease and its comorbidities [63]. They are hepatoprotective agents that are also useful to prevent or treat hepatic damages induced by chemicals [59].

### 5.2. Treatment of Atherosclerosis

**Figure 4:**
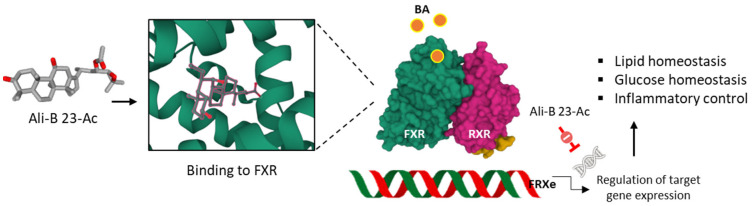
Anti-atherosclerosis activity of Ali-B 23-acetate. The compound binds to the FRX protein and displays an agonist activity which leads to the regulation of the expression of genes implicated in the control of inflammation, glucose, and lipid levels. Via this mechanism, Ali-B 23-acetate reduces atherosclerotic plaque.

Anti-atherosclerotic actions have been reported with Ali-A, its 24-acetate derivative and Ali-B 23-acetate. For the latter compound, the cholesterol-lowering effect is associated with an increase in FXR-BSEP signaling which leads to a reduction in liver cholesterol, hepatic lipolysis, and bile acids efflux. These cumulated effects concur in reducing damages of atherosclerosis [52]. In addition, the compound exerts anti-inflammatory effects, notably an inhibition of the production of inflammatory cytokines IL-12 (interleukin-12) and IFN-γ (interferon-γ), also contributing to the anti-atherosclerotic effect [64]. The capacity of Ali-B 23-acetate to markedly reduce the atherosclerotic plaque area and lipid accumulation is most likely the resultant of a cumulative effect implicating FXR agonism, anti-inflammatory effects and regulation of cholesterol synthesis and export (Figure 4). Indeed, Ali-B 23-acetate negatively regulates Acyl-CoA cholesterol acyltransferase 2 (ACAT2) in cultured Caco-2 cells and increases expression of ATP-binding cassette transfer proteins G5/G8 (ABCG5/G8) [65]. Multiple receptors and pathways contribute to the capacity of Ali-B 23-acetate to reduce accumulation of triglycerides and cholesterol, including the estrogen receptor α (ERα) to which the compound may bind directly, according to a molecular docking analysis [66]. However, other receptors have been proposed also. For example, the related compounds 16-hydroxy-Ali B 23-acetate and Ali-M 23-acetate have been predicted to bind, with a high affinity, to the liver X receptor β (LXRβ) on the basis of molecular docking and molecular dynamic simulations and to exert an agonist effect [67].

A distinct mechanism has been invoked to explain the anti-atherosclerotic activity of Ali-A. In this case, the compound activated the AMPK/SIRT1 signaling pathway, thereby inhibiting the formation of arterial plaques to block the progression of atherosclerosis [68]. A molecular docking study suggested that Ali-A could bind selectively to the catalytic region of AMPK, and, as such, the compound could activate the AMPK/acetyl-CoA carboxylase/SREBP-1c pathway, leading to reduced hepatic steatosis and improved liver function in a model of obese mice [69,70]. Ali-A can regulate lipid metabolism via different processes, including inhibition of inflammatory cytokine production. Its derivative Ali-A 24-acetate has been shown to inhibit the proliferation of aorta vascular smooth muscle cells induced by oxidized low-density lipoproteins, and to inhibit the expression of cyclin proteins (cyclin D1, E, p21, p27), also contributing to the reduction in atherosclerosis [71]. Ali-A 24-acetate can also inhibit activity of HMG-CoA reductase, presumably via a direct binding to HMG-CoA. This effect would also contribute to the observed reduction in total cholesterol [72]. Altogether, these different products, targets, and mechanisms contribute to the efficacy of AR to reduce hyperlipidemia and to combat atherosclerosis [73]. A recent network analysis pointed out the implications of 6 key targets regulated by AR (albumin, TNF, IL-1β, MMP9, and PPARα/γ) [74] whereas another study underlined the contribution of the FKBP/mTOR/SREBPs pathway and the direct binding of the active terpenoids to FK506-binding protein FKBP38 [10]. At least 9 triterpenes are considered to play a significant role in the lipid-lowering effect of AR (out of 87 triterpenes identified in the study) [75]. The complexity of the mechanism of action is not surprising. The natural medicine (mixture) is more potent than the individual compounds at regulating blood lipids, possibly due to synergistic effects between the compounds, with Ali-A and Ali-B being the most powerful lipid-regulating isolated products [76]. The best lipid-regulating effect has been obtained with a reconstituted mixture of Ali-A, Ali-B, and Ali-C 23-acetate in proportions of 3:1:1 [77].

### 5.3. Anti-Inflammatory Activity

**Figure 5:**
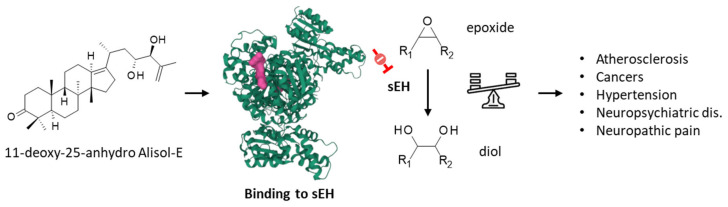
Binding of 11-deoxy-25-anhydro alisol-E to soluble epoxide hydrolase (sEH), the enzyme which converts epoxide molecules into diol molecules, as represented. The modulation of the epoxide/diol ratio is a mechanism by which the compound exerts activities against multiple pathologies, as indicated.

Soluble epoxide hydrolase (sEH) is a therapeutic target considered for the treatment of inflammation-based diseases, and various pathologies, including cardiovascular diseases and neuropsychiatric disorders [78,79]. A methanol extract of AR was shown to attenuate the decrease in epoxide hydrolase induced in rats intoxicated with bromobenzene [80]. The extracts contained protostane triterpenoids acting as inhibitors of sEH, such as alismanin B, 11-deoxy-25-anhydro-Ali-E (Figure 5), and 11-deoxy-Ali-B with IC_50_ values of 7.15, 3.40, and 5.94 μM, respectively. The compounds were predicted to bind to the active site of she, acting as competitive inhibitors [81]. More recently, two other compounds in the series were described shesEH inhibitors: 3β-hydroxy-25-anhydro-Ali-F and 3β-hydroxy-Ali-G (2), with IC_50_ values of 10.06 and 30.45 μM, respectively [82]. In contrast to Ali-F, Ali-A and Ali-B have not been described as sEH inhibitors. Ali-F and 25-anhydro–Ali-F are interesting compounds because they both behave as inhibitors of lipopolysaccharide-induced NO production in macrophage RAW 264.7 macrophages [83] and regulate the production of inflammatory cytokines, such as TNF-α, IL-1β, and IL-6, via the MAPK/STAT3/NFκB pathway [84]. sEH contributes to multiple human pathologies, chiefly inflammatory diseases but also cancer. Compounds such as Ali-E and Ali-F could be further exploited to design sEH-targeted drugs for the treatment of advanced liver cancers and other liver diseases.

Ali-B 23-acetate displays anti-inflammatory effects, apparently not via sEH inhibition, but through inhibition of the TLR4-NOX1/ROS signaling pathway. It also reduces production and infiltration of the same inflammatory cytokines as 25-anhydro-Ali-F. It suppressed expression of TLR4 and NOX2 and reduced the production of ROS [85]. Via these signaling pathways, the compound provides an anti-inflammatory action and maintains the integrity of the intestinal barrier as well [86,87]. Ali-B 23-acetate is an important contributor to the anti-inflammatory action of AR, but other protostane triterpenoids in the series also contribute, such as 16*S*,24*S*-dihydroxy-24-deacetyl-Ali-O and 16-oxo-11-anhydro-Ali-A, both characterized as anti-inflammatory agents [88].

### 5.4. Protection against Fibrosis and Kidney Injuries 

Renal fibrosis is an important component of chronic kidney disease (CKD), which affects more than 10% of the world’s population [89]. Ali-B 23-acetate can attenuate the progression of CKD, at least in a rat model. An oral treatment with the compound (5–10 mg/kg) has been shown to inhibit expression of mRNA and proteins implicated in CKD pathogenesis, such as collagen I, fibronectin, vimentin, α-smooth muscle actin, and fibroblast-specific protein-1. Interestingly, the compound protected against renal fibrosis in part by re-establishing dysbiosis of the gut microbiome and regulating blood pressure (reducing hypertension). It did so by repressing the Wnt/β-catenin signaling pathway implicated in the activation and proliferation of renal fibroblasts [90]. The renin–angiotensin system and the Wnt/β-catenin axis play a role in kidney inflammation and fibrosis, and thus contribute to CKD. A treatment with Ali-B 23-acetate could reduce disease progression [91]. The activity is not specific to Ali-B 23-acetate, as the related product Ali-F 25-O-methyl has been shown also to attenuate tubulo-interstitial fibrosis, even if a distinct biochemical pathway (inhibition of TGF-β-mediated Smad3 phosphorylation) was invoked in that case [92].

The capacity of Ali-B 23-acetate to regulate the gut microbiome is beneficial not only for kidneys, but also for the gastro-intestinal tract and for immune regulation in general. The gut microbiota plays a role in metabolic diseases, cancers, and other pathologies. The probiotic regulation with Ali-B 23-acetate can reduce organ fibrosis (such as hepatic and renal fibrosis) but also colitis (associated with colon cancer) [86,93,94]. Ali-B 23-acetate and the other protostane triterpenoids of AR behave as regulators of the gut microecology. They can equilibrate the gut microflora, notably in cases of metabolic diseases and atherosclerosis [95,96].

### 5.5. Antioxidant Activity and Neuro-Protection

In the alisol series, the derivative Ali-A 24-acetate is an interesting cell-protective compound that is useful to limit brain damages. Both Ali-A and -B can cross the blood–brain barrier. Their acetate derivatives can also distribute in the brain tissue, although they preferentially accumulate in the intestine, stomach, liver, and kidneys, after oral administration [34]. Nevertheless, Ali-A 24-acetate has been shown to protect brain microvascular endothelial cells (BMEC) from damages induced by oxygen-glucose deprivation [97]. It exerts anti-apoptotic effects in this cell system [98] and also reduces apoptosis of neurons, via up-regulation of the expression of phosphorylated PI3K and AKT [99]. The blockade of thee PI3K/Akt/mTOR signaling pathway is important to the mechanism of action of AR and specifically to Ali-A 24-acetate, not only providing a protection against neuronal damages but also essential to the anticancer action of the extract and the product, as discussed thereafter [100].

### 5.6. Bone Preservation and Prevention of Osteoporosis

**Figure 6:**
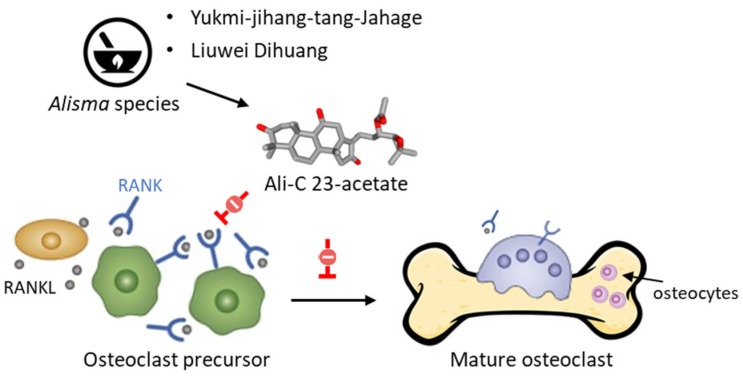
Inhibition of RANKL-induced osteoclast differentiation and function by Ali-C 23-acetate (AC23A). The compound would act selectively on osteoblast precursors, rather than mature osteoblasts, by inhibiting RANKL-induced osteoclastogenesis. The compound blocks phosphorylation of JNK and reduces expression of osteoclastogenic mediators, such as TRAP, c-Fos, MMP9, NFATc1, and cathepsin K [104]. AC23A can be found in phytomedicines made from Alisma rhizoma, such as Liuwei Dihuang and Yukmi-jihang-tang-Jahage, both used to treat osteoporosis.

The acetate derivatives of Ali-A and Ali-B have been shown to protect against a decrease in bone mass. As a matter of fact, AR and different TCM preparations containing *Alisma* species (*A. orientale*, *A. plantago-aquatica*, *A. canaliculatum*) are used to regulate bone resorption, such as Yukmi-jihang-tang-Jahage [101] and Liuwei Dihuang [102]. Both Ali-A 24-acetate and Ali-C 23-acetate display anti-osteoporotic effects [103]. The latter compound is a potent inhibitor of osteoclastogenesis, acting via a blockade of RANKL-induced osteoclast differentiation and function (Figure 6) [104].

Ali-A 24-acetate also functions as an inhibitor of osteoclastogenesis. It inhibits RANKL-mediated osteoclast differentiation by downregulating the expression of nuclear factor of activated T cell 1 (NFATc1), which is an important regulator of osteolysis [105]. In an animal model of postmenopausal osteoporosis (ovariectomized mice), a daily administration of Ali-A 24-acetate was shown to prevent bone loss, inhibiting activity of the bone resorption marker TRAP and restoring the proportion of Treg and Th17 immune cells [106]. RANKL-mediated effects have been also reported with Ali-B, e.g., inhibition of osteoclast formation and suppression of hypercalcemia [107].

### 5.7. Anticancer Activity

There is ample experimental evidence to support the activity of alisol derivatives against cancer cell proliferation and tumor progression (Table 1). The main compounds Ali-A/-B and their acetate derivatives have been used in different tumor models, the other alisol derivatives have been used less frequently. However, Ali-F 24-acetate was shown to reverse multidrug resistance, thereby promoting nuclear accumulation of the cytotoxic drug doxorubicin and apoptosis of MCF-7 breast cancer cells [108]. A comparable reversal of P-glycoprotein-mediated multidrug resistance was evidenced with Ali-B 23-acetate [109]. There are not many studies comparing the anticancer efficacy of the different products, and the cell models used vary significantly from one study to another. Ali-A has revealed a marked capacity to reduce proliferation and invasion/migration of breast cancer cells [110,111], whereas Ali-B has shown a potent activity against hepatocellular carcinoma cells [112,113] and other models (Table 1). It is not easy to compare the potency of the products. However, there are common characteristics between the compounds, such as their capacity to induce G_0_/G_1_ cell cycle arrest and to trigger apoptosis via the mitochondrial pathway (Table 1).

The PI3K/Akt/mTOR pathway is central to the mechanism of action of Ali-A/-B [100] but it is not the sole pathway implicated in the anti-proliferative and antimetastatic action of the natural products. A recent study pointed out the major implication of the Wnt/*β*-catenin axis in the mechanism of action of Ali-B 23-acetate in liver cancer [123]. The compound functioned as an inhibitor in SMMC-7721 and MHCC97 cancer cells to promote the anticancer action of the cardiac glycoside bufalin (an active ingredient of the traditional Chinese medicine Chansu) [123]. The same pathway has been invoked to explain the anti-fibrotic action of the product [91]. The complexity of the signaling pathways involved in the mechanism of action of natural products is not surprising, it is frequently the case with terpenoids [124].

**Figure 7:**
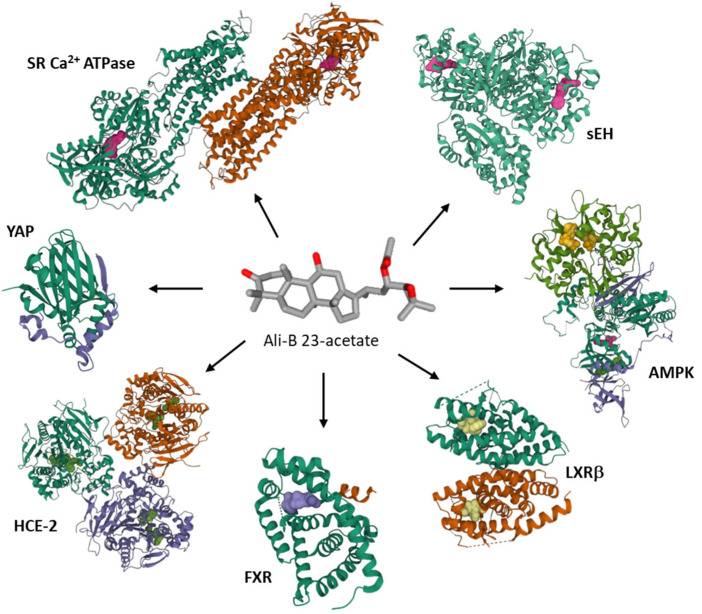
Proposed protein targets for Ali-B 23-acetate. Molecular modeling studies have evaluated binding of the compound to soluble epoxide hydrolase (sEH) [81,82], 5’-adenosine monophosphate-activated protein kinase (AMPK) [70], liver X receptor β (LXRβ) [67], farnesoid X receptor (FXR) [51], human liver carboxylesterase 1 (HCE-2) [126], Yes-associated protein (YAP) [115], sarcoplasmic/endoplasmic reticulum Ca^2+^ ATPase (SR Ca^2+^-ATPase) [125]. The protein models shown correspond to the PDB structures 4HAI for sEH, 7MYJ for AMPK, 6K9H for LXRβ, 6HL1 for FXR, YA4 for HCE-1, 3KYS for YAP, 1VFP for SR Ca^2+^-ATPase.

At the molecular level, several protein targets have been evoked to account for the anticancer activity of Ali-A/-B, essentially on the basis of molecular modeling studies, such as the transcriptional coactivator YAP which could interact with Ali-A [115], the sarcoplasmic/endoplasmic reticulum Ca^2+^ ATPase which can bind Ali-B [125], the soluble epoxide hydrolase [81], the farnesoid X receptor protein [51], human liver carboxylesterase 1 (HCE-2) [126], and hormone receptors [127] (Figure 7). Occasionally, non-protein targets have been mentioned as well, such as DNA. Both Ali-A and Ali-B have been docked onto DNA and shown to compete with DNA intercalation of the fluorescent dye ethidium bromide [128]. The c-myc DNA sequence has been considered as a potential target for Ali-A/-B acetates and the compounds’ mixture [129]. The hypothesis has not been validated experimentally at present. These terpenoids are multi-targeted products.

There are many published in vitro studies with Ali-A/-B using different cell lines, but relatively little evidence of antitumor activity in vivo. A study reported the capacity of Ali-B 23-acetate to reduce growth of a subcutaneously implanted hepatocellular carcinoma tumor in mice, but it was a tumorigenesis experiments with tumor cells treated with the compound (at 30 μM for 24 h) prior to grafting the tumor cells to mice. The compound delayed the growth of the tumor in mice [117]. It is not an anticancer treatment *sensu stricto*. There is a lack of robust evidence of an antitumor action of alisols in xenograft models.

### 5.8. Anti-Allergic Activity

In traditional medicine, the dried rhizomes of *Alisma orientale* are used to treat allergic reactions. The capacity of AR extracts to inhibit antibody-mediated allergic reactions has been recognized more than 25 years ago and, at that time, the anti-allergic contribution of compounds such as Ali-A and Ali-B was already mentioned [130]. Ali-B 23-acetate has been characterized as a major inhibitor of 5-lipoxygenase-catalyzed leukotriene C_4_ production in rat basophilic leukemia RBL-1 cells [131]. This compound reduces IgE/antigen-induced degranulation of mast cells and ameliorates allergic reaction [132].

### 5.9. Antibacterial and Antiviral Effects

Ali-A/-B are not highly potent antibacterial agents per se. They showed little, if any, antimicrobial effects against pathogenic bacteria, such as *Bacillus subtilis*, *Escherichia coli*, and *Staphylococcus aureus*. Only minor effects have been reported with the derivatives such as Ali-G and 25-anhydro-Ali-F [83]. Both Ali-A 24-acetate and Ali-B 23-acetate have been found to be active against antibiotic-resistant strains of *S. aureus* and *E. coli* [133] but their antibacterial effects have not been further investigated. However, Ali-B 23-acetate is a useful regulator of gut microbiota, capable of reducing the expansion of pathogenic bacteria (such as *Klebsiella*, and *Citrobacter*) and increasing growth of beneficial bacteria (such as *Bacteroides*, and *Lactobacillus*) [94].

Similarly, Ali-A/-B are not highly effective antiviral compounds, although there are occasional reports of alisol derivatives being applied effectively against specific viruses, notably such as limiting the secretion of hepatitis B virus (HBV) surface antigen with a tri-acetate derivative of Ali-A and some derivatives [134,135]. Based on this observation, acylated derivatives of Ali-A have been designed, leading to the identification of tetra-acetyl Ali-A as an orally bioavailable derivative with satisfactory pharmacokinetic properties, such as being active against HBV in cultured HepG 2.2.15 cells [136]. However, no further development has been reported for this derivative.

## 6. Discussion and Perspectives

Ali-A/-B and their acetate derivatives are the most abundant triterpenes present in AR. They can serve as control markers to evaluate the quality of Zexie phyto-prepaprations of the Chinese Pharmacopoeia [75,137]. Ali-B and Ali-B 23-acetate are abundant in the plant roots, but also in the leaves, scapes, and inflorescences [138]. Ali-A is one of the quality markers which can be used to assess the quality of the TCM prescription Qiliqiangxin capsule (QLQX), used to treat chronic heart failure [18], whereas Ali-B 23-acetate is a quality marker for the Hugan Qingzhi formula used to treat nonalcoholic fatty liver disease [12]. However, the bioactivity not only depends on the quality of the phyto-preparation and the alisol content, but also on the respective proportions of the bioactive molecules, notably the ratio of Ali-A 24-acetate, Ali-B, and Ali-B 23-acetate [139]. There are robust methods to precisely quantified these compounds in AR extracts and methods to optimize their extraction also from AR, notably for Ali-B 23-acetate which is arguably the most potent and most abundant compound in the tubers [140].

As presented above, the main four alisol derivatives displays a large range of pharmacological effects. They have been tested in multiple models, either alone or within AR extracts, and occasionally using reconstituted mixtures. Evidence of activity has been reported in most cases, but the degree of efficacy is variable. On the one hand, the lipid-lowering effects, anti-inflammatory, anti-atherosclerosis, and antidiabetic activities have been firmly established, using both in vitro and robust in vivo models. Nobody can dispute the fact that the four compounds function as regulators of lipid levels in blood [64,95]. The capacity of both Ali-B and Ali-B 23-acetate to attenuate and to protect from non-alcoholic steatohepatitis (NASH) has been well-established in mice models with convincing data [50,54]. In contrast, we can challenge the anticancer activity reported with the same compounds, despite the multiplicity of in vitro studies with experimental cell lines. Evidence of a real anticancer effect, with xenograft models of tumors, are lacking and the potency of the compounds appear limited, at least when they were tested alone. There is no need to deny the anti-proliferative effects and pro-apoptotic activities evidenced in a variety of cell models (Table 1), but one must admit that at present there is no solid evidence of an antitumor effect in vivo with Ali-A or Ali-B in a xenograft model. There is a need to better characterize the anticancer effects of the compounds in xenografts and to consider further the combination of these lipid modulators with chemo- and immunotherapeutic drugs. Another aspect, perhaps also insufficiently exploited at present, is the capacity of AR and its constituents to regulate the microbiota. The probiotic effects of Ali-A 23-acetate evidenced recently could be useful to combat various pathologies affecting the liver and gastro-intestinal tract [86,94,95]. Other properties are secondary, such as the mild antibacterial and antiviral effects, not sufficiently robust to be exploited in terms of drug development.

The toxicological profile of the alisols needs to be properly defined. There is no published study formally evaluating the toxicity of these natural products, but there are reports on the toxicity of AR in a clinical setting and about the renal toxicity of *Alisma orientalis* [141]. In a rat model, renal damages have been observed after a long treatment with AR (on day 180) [142]. The dosing of AR is known to be important to control the diuretic and anti-diuretic effects observed with the products [36,143]. The molecular mechanism whereby Ali-A 24-acetate and Ali-B 23-acetate induce cell death (autophagy and then apoptosis) of human renal proximal tubular HK-2 cells has been detailed. The central point was the engagement of the PI3K/Akt/mTOR signaling pathway [144]. The two compounds contribute to the nephrotoxicity of AR, together with other terpenoids present in the extract [42]. A careful analysis of their toxicity profile is needed, all the more than the compounds bind strongly to blood proteins, such as hemoglobin [145] and they also distribute well in fat tissues [34], benefiting from an enterohepatic circulation [146].

## 7. Conclusions

**Figure 8:**
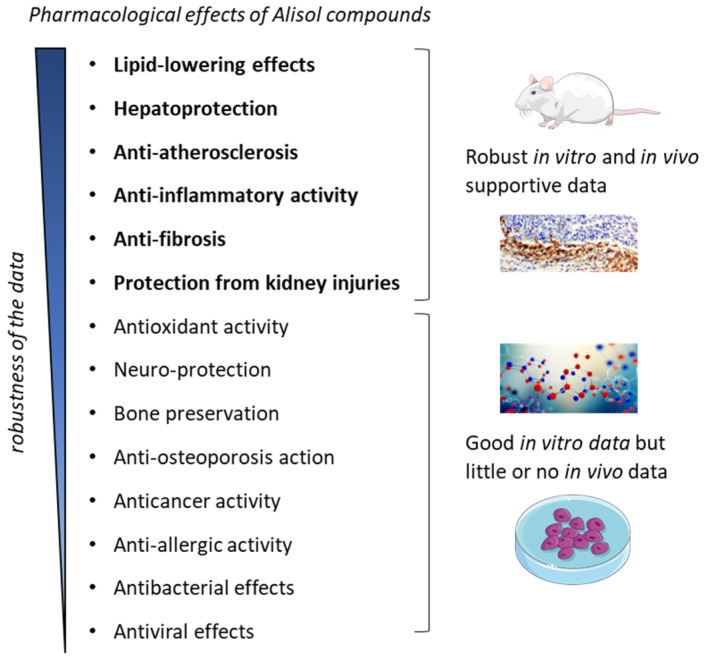
Pharmacological effects reported with alisol derivatives. The effects can be separated into two categories: the effects benefiting from robust in vitro and in vivo data obtained with animal models representative of the pathology (indicated in bold) and the effects evidenced essentially using in vitro data, with purified molecular systems and/or laboratory cell lines.

Altogether, the pharmacological effects of Ali-A/-B and their acetate derivatives can be separated in two groups: (1) those with robust in vitro and in vivo data obtained with animal models representative of the studied human pathology; (2) those benefiting essentially, if not exclusively, from in vitro data, and for which additional supportive data are needed (Figure 8). The lipid-lowering, cell protective, and anti-inflammatory effects of Ali-A/-B have been fully characterized (group 1). In contrast, the anti-proliferative and pro-apoptotic activities of the compounds have been well evidenced at the cellular level, but further evidence to support an antitumoral effect in vivo are absolutely needed (group 2). The diverse TCM and phytomedicines containing AR and used to treat cardiovascular and inflammatory diseases can benefit from the pharmacological effects of the alisol compounds. The use of these natural medicines to prevent atherosclerosis and fibrosis can be encouraged.

## Figures and Tables

**Table 1:** Anticancer activity of alisol derivatives.

Compounds	Cancer Cell Types (Cell Lines)	Observed Effects	Reference
Ali-A	Colorectal cancer cells (HCT-116, HT-29)	Cell growth inhibition. Induction caspase-dependent apoptosis and pyroptosis. Repression of cell migration, through down-regulation of N-cadherin, up-regulation of E-cadherin.	[114]
Ali-A	Nasopharyngeal carcinoma cells (C666-1, HK1)	Cell growth inhibition, G_0_/G_1_ cell cycle arrest with down-regulation of cyclins D1/E1 and CDK2/4. Inhibition of cell migration, with down-regulation of MMP2/9. Binding to and phosphorylation of the transcriptional coactivator YAP.	[115]
Ali-A	Breast cancer cells (MDA-MB-231)	Inhibition of cell proliferation, G_1_ cell cycle arrest, induction of apoptosis and autophagy. Induction of ROS and DNA damage. Blockade of NFκB and PI3K/Akt/mTOR pathways. Suppression of cell migration and invasion, via inhibition of MMP2/9.	[110,111]
Ali-B	Melanoma cells (B16)	Cell growth inhibition. Downregulation of MITF, via suppression of CREB and activation of ERK.	[116]
Ali-B	Breast cancer cells (MDA-MB-231)	Inhibition of cell growth; caspase-dependent mitochondrial apoptosis; accumulation of ROS; downregulation of p-AKT, p-p65, and p-mTOR.	[117]
Ali-B 23-acetate	Ovarian cancer cells (A2780, HEY)	G_1_ cell cycle arrest, down-regulation of CDK4/6, cyclin D1. Up-regulation of Bax/Bcl-2 ratio and induction of endoplasmic reticulum stress through IRE1 signaling. Suppression of cells migration and invasion, with inhibition of MMP-2/9.	[118]
Ali-B 23-acetate	Colon cancer cells (SW620, HCT116)	Cell growth inhibition, induction of apoptosis and autophagy, dependent on the production of ROS and phosphorylation of JNK.	[119]
Ali-B 23-acetate	Lung cancer cells (A549, NCI-H292)	Cell growth inhibition, induction of mitochondrial apoptosis, generation of ROS. Reduced phosphorylation of AKT, PI3K, and mTor. Inhibition of cell migration/invasion.	[88,120]
Ali-B 23-acetate	Gastric cancer cells (AGS, SGC7901)	Inhibition of cell proliferation, induction of mitochondrial apoptosis, generation of ROS. Regulation of MAPK activation.	[121,122]
Ali-B 23-acetate	Hepatocellular carcinoma cells(SK-HEP-1, HepG2, SMMC-7721, MHCC97)	Cell growth inhibition, G_1_ cell cycle arrest, induction of apoptosis. Inhibition of cell migration. Reduction in tumorigenesis (in vivo) with pretreatment of the cells in vitro (before grafting). Repression of mTOR pathway-related proteins. Inhibition of the Wnt/β-catenin pathway.	[112,113,123]

## Data Availability

Not applicable.
